# Validating indicators of CNS disorders in a swine model of neurological disease

**DOI:** 10.1371/journal.pone.0228222

**Published:** 2020-02-19

**Authors:** Vicki J. Swier, Katherine A. White, David K. Meyerholz, Aude Chefdeville, Rajesh Khanna, Jessica C. Sieren, Dawn E. Quelle, Jill M. Weimer

**Affiliations:** 1 Pediatrics and Rare Diseases Group, Sanford Research, Sioux Falls, South Dakota, United States of America; 2 Department of Pathology, University of Iowa Carver College of Medicine, Iowa City, Iowa, United States of America; 3 Department of Pharmacology, College of Medicine, University of Arizona, Tucson, Arizona, United States of America; 4 Graduate Interdisciplinary Program in Neuroscience; College of Medicine, University of Arizona, Tucson, Arizona, United States of America; 5 Department of Radiology and Biomedical Engineering, University of Iowa, Iowa City, Iowa, United States of America; 6 Department of Neuroscience and Pharmacology, University of Iowa, Iowa City, Iowa, United States of America; 7 Department of Pediatrics, Sanford School of Medicine, University of South Dakota, Sioux Falls, South Dakota, United States of America; Sichuan University, CHINA

## Abstract

Genetically modified swine disease models are becoming increasingly important for studying molecular, physiological and pathological characteristics of human disorders. Given the limited history of these model systems, there remains a great need for proven molecular reagents in swine tissue. Here, to provide a resource for neurological models of disease, we validated antibodies by immunohistochemistry for use in examining central nervous system (CNS) markers in a recently developed miniswine model of neurofibromatosis type 1 (NF1). NF1 is an autosomal dominant tumor predisposition disorder stemming from mutations in *NF1*, a gene that encodes the Ras-GTPase activating protein neurofibromin. Patients classically present with benign neurofibromas throughout their bodies and can also present with neurological associated symptoms such as chronic pain, cognitive impairment, and behavioral abnormalities. As validated antibodies for immunohistochemistry applications are particularly difficult to find for swine models of neurological disease, we present immunostaining validation of antibodies implicated in glial inflammation (CD68), oligodendrocyte development (NG2, O4 and Olig2), and neuron differentiation and neurotransmission (doublecortin, GAD67, and tyrosine hydroxylase) by examining cellular localization and brain region specificity. Additionally, we confirm the utility of anti-GFAP, anti-Iba1, and anti-MBP antibodies, previously validated in swine, by testing their immunoreactivity across multiple brain regions in mutant *NF1* samples. These immunostaining protocols for CNS markers provide a useful resource to the scientific community, furthering the utility of genetically modified miniswine for translational and clinical applications.

## Introduction

Animal models are essential tools for studying the underlying mechanisms of disease as well as providing a platform for preclinical research and drug discovery. Historically, rodents have been one of the primary model systems for studying disease and driving drug discovery, largely due to the widespread availability of well-described and validated reagents for use in these model organisms. However, there are increasing instances where rodent models either fail to recapitulate aspects of human disease [1], or where treatments that are efficacious in a rodent model fail to translate to viable human therapies [2]. This has led to development of large animal models of disease, such as genetically modified swine, that may bridge the gap between basic and translational science by offering disease models that are more similar to humans anatomically, genetically, physiologically, and metabolically [3–6]. This increased similarity is especially important when studying neurological disorders. Compared to human anatomy, the mouse brain lacks gyri and sulci in the cerebrum and has much less white matter [7]; physiologically, mice also differ in immune receptors, cell types, and signaling pathways [8]. These anatomical and physiological differences found in the rodent systems cannot recapitulate human disease. Hence, successful genetically modified miniswine models have been established to study a number of human diseases including atherosclerosis, cancer, ataxia telangiectasia, cystic fibrosis, and neurofibromatosis type 1 [3, 6, 9, 10]. However, while these large animal systems can better recapitulate many of the hallmarks of human disease, there are limited tools and reagents that have been well described and validated in these models, limiting the translatable application of these models. Herein, to provide this resource to the scientific community, we validate a number of antibodies relevant to the study of the brain and neurological disorders using immunohistochemistry in porcine brain tissue.

As some antibody targets are predominantly reactive in a disease state, here we use a recently developed miniswine model of neurofibromatosis type 1 (NF1) to validate a number of CNS cell-specific antibodies [3]. Neurofibromin, the protein encoded by *NF1*, is expressed in astrocytes, oligodendrocytes and neurons of the brain [11]. Heterozygous mutations in *NF1* alter the expression of neurofibromin, a protein best known for its function as a GTPase-activating protein for RAS, leading to tumor predisposition throughout the nervous system. As the level of neurofibromin is altered in CNS cells of patients with NF1, a host of CNS-specific impairments occur, requiring the need for multiple neurologically relevant antibodies to understand the complexities of this neurological disease in a swine model. We validate and explore the expression of antigens implicated in glial inflammation, oligodendrocyte differentiation, neuronal signaling, and nociceptive function. Taken together, we provide a powerful set of tools to researchers modeling neurological dysfunction in porcine models of disease [3].

## Materials and methods

### Animal tissue

All miniswine were maintained at Exemplar Genetics under an approved IACUC protocol. All mice were maintained in an AAALAC accredited facility in strict accordance with National Institutes of Health guidelines, and studies were approved by the Sanford Institutional Animal Care and Use Committee (USDA License 46-R-0009). Animals were euthanized by an injection of Euthasol (390 mg/ml Pentobarbital, 50 mg Phenytoin per ml). Whole and half hemispheres of miniswine brain were fixed in 10% formalin for 3 weeks, and whole mouse brain was fixed in 10% formalin for 24 hours.

### Tissue microarray

Regions from formalin-fixed cortex (CTX), cerebellum (CB), hippocampus (HPC), thalamus (THAL), corpus callosum (CC), and cerebral aqueduct (CGG) of a 15-month old, male *NF1* miniswine [3] and 15-month old, female wild-type miniswine were isolated and placed in tissue cassettes. These regions were selected due to their relevance to neurologic disease (including NF1) in relation to macrocephaly (CC) [12], white matter abnormalities (CTX, CB, and CC) [13], brain lesions (CB and THAL) [14], abnormal physiology (HPC) [15], and aqueductal stenosis (CGG) [16]. The tissue cassettes were processed, embedded in paraffin, and sections were H&E stained. Subsections of interest were marked on each slide and a circular biopsy was taken from the paraffin block that matched the marked region. The paraffin biopsies were placed into a tissue microarray mold and re-embedded in paraffin to create a paraffin microarray block. Sections of the paraffin microarray block were cut and floated onto slides.

### Strategy for antibody selection

Details regarding each of the antibodies used in this study are listed in Table 1. When possible, we selected antibodies predicted to work in swine or constructed with a porcine immunogen, however, very few of these antibodies exist. Therefore, we primarily selected antibodies known to react in multiple mammalian species (such as mouse, rat and human), as the degree of homology between swine and the aforementioned mammalian proteins is fairly high (89–100% similarity) [17], especially for evolutionarily conserved genes.

**Table 1:** Description, dilution and cellular localization of each antibody tested.

Antibody	Company/Number	Dilution	Reacts with Species	Positive Control	Raised In	Target	Cell localization
GFAP	Dako 70334	1:1000	H,M,R, D,Ch,B,Sh*	Meyerholz et al. 2017	rabbit	Mature Astrocyte	cytoplasm, intermediate filaments
CD68	abcam ab125212	1:400	H,M,R	Cerebral cortex	rabbit	Active microglia	membrane
IBA1	BioCare Medical 290	1:500	H,M,R	Meyerholz et al. 2017	rabbit	All Microglia	cytoplasm
NG2	abcam ab129051	1:250	H,M,R	Cerebral cortex	rabbit	Olig. Progenitor (immature Olig.)	membrane, cytoplasm
O4	R&D Systems MAB1326-SP	1:400	H, M, R, Ch	White matter	mouse	Oligodendrocyte	membrane
Olig-2	Millipore ab9610	1:500	H,M,R	Cerebral cortex	rabbit	All Oligodendrocytes	nucleus and cytoplasm
MBP	Millipore MAB386	1:100	H,M,R,Ch,B,Sh,Rb,Gp	Meyerholz et al. 2017	rat	Mature Oligodendrocytes	membrane, cytoplasm
Doublecortin	SantaCruz sc-28939	1:50	H,M,R*	Cerebral cortex	rabbit	Immature Neurons	cytoplasm
GAD67	BD BioSciences 611604	1:50	R	Cerebral cortex	mouse	GABAergic Neurons, dendrites, axons	cytoplasm, membrane, nucleus
Tyrosine Hydroxylase	Millipore AB152	1:500	H, R, M, Fe, Ft, Sqd, Dr, Ml	Hypothalamus	rabbit	Dopaminergic Neuron	cytoplasm
Myelin PLP	abcam ab28486	1:500	H, M, R, Rb*	Cerebral cortex	rabbit	Mature Oligodendrocytes	membrane

Data presented as antibody, company, catalog number, dilution, reaction with species, the citation or tissue type that was used as positive control, the host animal, target of the antibody, and cellular localization of each antibody examined. Target and cellular location were either cited from the antibody data sheet (provided by the company) or obtained from the Human protein atlas, https://www.proteinatlas.org/.

* indicates predicted to react in swine. Abbreviations are H (human), M (mouse), R (rat), D (drosophila), Ch (chicken), B (bovine), Sh (sheep), Rb (rabbit), Gp (Guinea pig), Fe (feline), Ft (ferret), Sqd (squid), and MI (mollusc).

To support the specificity of these antibodies in miniswine, we also ran an NCBI standard protein BLAST alignment on the immunogen that was used to develop the antibody (if that information was available from the vendor) to non-redundant protein sequences of Sus scrofa (domestic swine). In most cases, we found greater than 85% identity to Sus scrofa protein sequences, except in the case of CD68 at 69% identity and TRPV1 at 68% identity (S1 Table).

Specificity of antibody binding was assessed through anticipated regional and cellular localization, and publication searches on the targeting of the antibody. Specifically, we searched Antibodypedia (www.antibodypedia.com), the Antibody Registry (antibodyregistry.org), vendor websites, and our previous publications to determine how these antibodies had been validated in other species (S1 Table) [18–20]. As many of these antibodies had been previously validated in mice, we searched the mouse brain tissue atlas (https://www.proteinatlas.org/) (28) and the gene expression database at the mouse genome informatics website (http://www.informatics.jax.org) (29) to identify immunopositive brain regions for those antigens that were not listed in the aforementioned antibody registries or previous publications. Hence, we choose coronal brain sections from 3-week-old, C57Bl/6J wild-type mice as our positive controls. The mouse tissues were immunolabeled alongside the miniswine tissue, to verify the proper reactivity, localization, and expression of the antibody in question.

### Immunohistochemistry on paraffin sections

Paraffin tissue arrays on slides were deparaffinized in xylene, rehydrated in ethanol, and rinsed in double distilled water. Antigen retrieval was performed at 90C for 20 minutes using sodium citrate buffer, pH 6. Then, slides were rinsed in 1xTBST, endogenous peroxidases were blocked in Bloxall^™^ (Vector Laboratories, Burlingame, CA) for 10 minutes, and rinsed again in 1xTBST. *For antibodies raised in rabbit*, blocking serum from an ImmPRESS^™^ HRP Anti-Rabbit IgG (Peroxidase) Polymer Detection Kit (Vector Laboratories) was incubated on slides for 20 minutes at room temperature (RT). Slides were drained and the primary antibody was incubated on the slides at 4C overnight. Negative controls without primary antibody were run in parallel with normal host IgG. Slides were rinsed in 1xTBST, and ImmPRESS^™^ (Peroxidase) Polymer was incubated on slides for 30 minutes at RT. Slides were rinsed in 1xTBST and 3,3’-diaminobenzidine (DAB) from Vector laboratories was added to the slides for 2 to 10 minutes until the DAB activation occurred. Slides were then washed with DI water, stained with Mayer’s hematoxylin, washed with running tap water, dipped in 0.25% Lithium carbonate, rinsed in DI water, dehydrated with ethanol, cleared with Xylene and mounted with DPX mounting media. *For antibodies raised in mouse*, an ImmPRESS^™^ HRP Anti-Mouse IgG (Peroxidase) Polymer Detection Kit (Vector Laboratories) was used, followed by the described DAB staining. *For antibodies raised in rat*, 1% goat serum with Triton was used as a blocking solution for at least 1 hour at RT and a Goat Anti-Rat IgG H&L (HRP) (Abcam ab97057) was used as a secondary and incubated for 1.5 hours at RT before continuing with the described DAB staining. Tissue sections were imaged with an Aperio Versa slide scanner (Lecia Biosystems Inc, Buffalo Grove, IL). All antibodies were subjected to the same protocol. To determine optimal dilutions of each antibody, and to insure reproducibility of the immunostaining, immunohistochemistry was performed for each antibody on at least two separate occasions. Concentration was adjusted based on level of background and non-specific immunostaining. Examples of dilutions tested included 1:250 to 1:1000 in the GFAP antibody and 1:100 to 1:500 in the MBP antibody.

To identify positive immunohistochemical staining to antibodies in Table 1, that differed from non-specific immunolabeling or background staining, we defined ‘positive immunostaining’ as an immunoreaction that was specific to the tissue and cellular localization of the antigen as determined in previous publications (found in the Antibody Registry, vendor websites, and our previous publications). Negative immunohistochemical staining was defined as the absence of an immunoreaction to tissues and cells that do not have that antigen.

### Immunohistochemistry on free-floating sections

Brain tissues from a 14-month old female *NF1* miniswine and a 20-month old male *NF1* miniswine were fixed in 10% formalin for 3 weeks. After fixation, the brains were cryoprotected in 30% sucrose and 45 μm frozen sections were cut on a freezing microtome. Free-floating sections were placed in 10 mM sodium citrate solution (pH 8.5) preheated to 80C in a water bath. The temperature was maintained at 80C while the sections incubated for 30 minutes [21]. Sections were then allowed to cool to room temperature, rinsed in 1xTBS, and stained immunohistochemically following previously published procedures [22]. The following antibodies were subjected to the same protocol: GFAP, CD68, Olig2, doublecortin, GAD67 and tyrosine hydroxylase. To determine optimal dilutions of each antibody, and to insure reproducibility of the immunostaining, immunohistochemistry was performed for each antibody on at least two separate occasions. The final dilutions used were 1:16000 for GFAP; 1:1:1000 for CD68; 1:2000 for Olig2; 1:250 for doublecortin; 1:1000 for GAD67; and 1:4000 for tyrosine hydroxylase.

### Dorsal root ganglia (DRG) immunostaining

Miniswine DRGs were fixed in 4% PFA for 24 hours, cryoprotected in 30% sucrose (m/v) in PBS for 48 hours and frozen at -70°C in 2-methylbutane chilled with dry ice. Samples were cut into 20μm-thick sections. Sections were subsequently blocked in 3% BSA, 0.1% Triton X-100 in PBS for 1 hour at room temperature then incubated with primary antibodies (anti-TRPV1, Neuromics GP14100; anti-CGRP, Abcam ab36001) diluted in blocking solution overnight at +4°C. After 3 washes in PBS, slides were incubated with secondary antibody diluted at 1/1000 in blocking solution for 2 hours at room temperature, washed and counterstained with DAPI. For the negative control, primary antibodies were omitted. Images were acquired on an Axio Imager 2 (Zeiss), using a 10X objective controlled by the Zen software (Zeiss).

## Results

### Glial marker immunolabeling in miniswine brain samples

**Fig 1:**
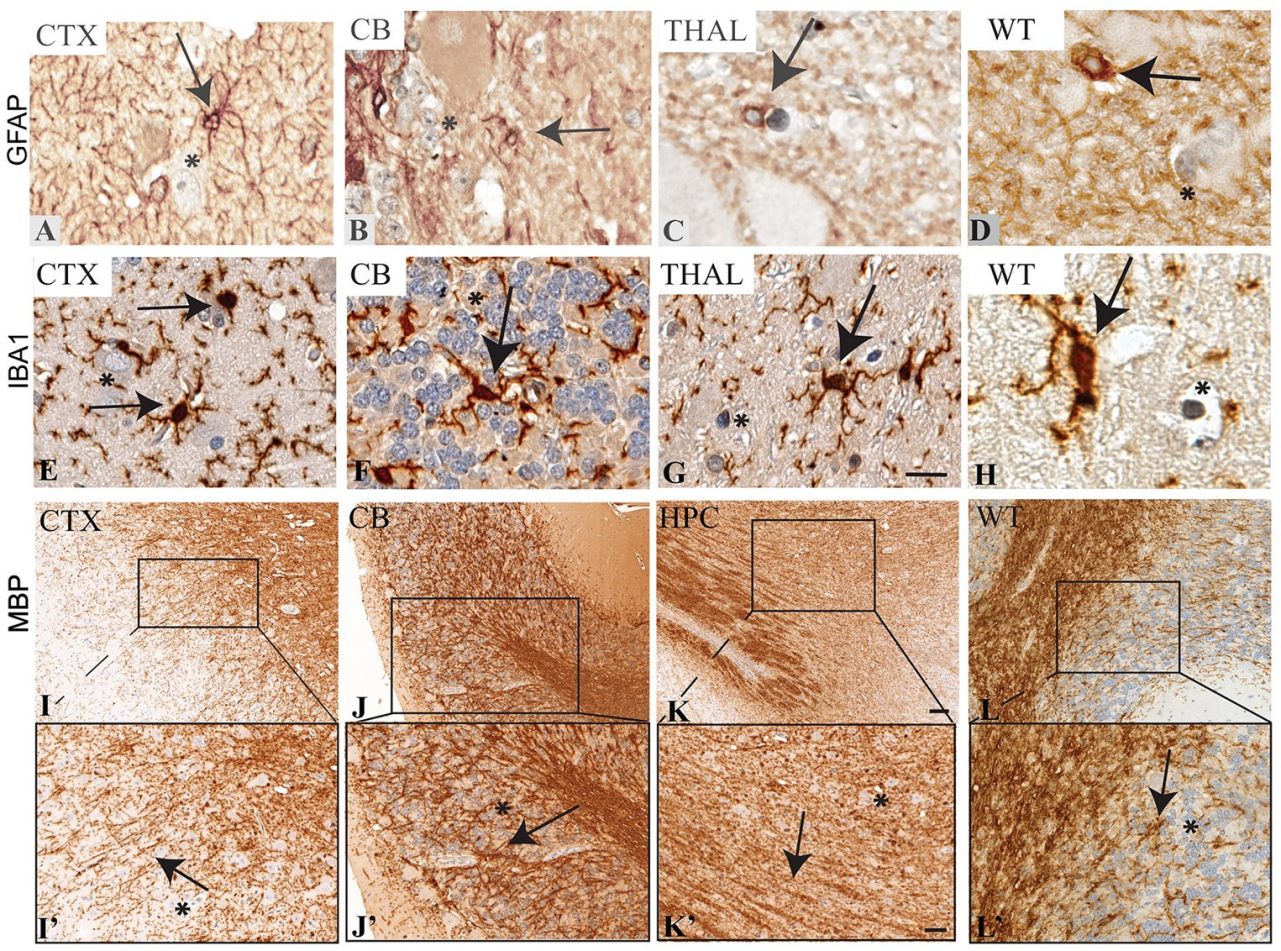
Immunolabeling of known markers in various regions of *NF1* mutant miniswine brain. Brain regions are identified as cortex (CTX), cerebellum (CB), thalamus (THAL), and hippocampus (HPC). A-D: GFAP+ expression in the cortex, cerebellum, and thalamus, localized to the membrane and filaments of astroyctes. Arrows indicate classic, star-shaped morphology of astrocytes. E-H: Iba1+ expression in the cortex, cerebellum, and thalamus, localized to the cytoplasm of microglia. Arrows indicate both ramified and ameboid-like microglia. Scale bar: 50μm. I-L: MBP+ expression in the cortex, cerebellum, and hippocampus, localized to the membrane and cytoplasm of mature oligodendrocytes. Scale bar: 500μm. I’-L’: Enhanced magnification of panels I-L. Scale bar: 200μm. Asterisks indicate lack of immunolabeling in neurons, as expected.

The following markers (GFAP, IBA1, and MBP) have been validated in the cerebrum of wild-type swine previously [23]. However, these antibodies have not been validated in other areas of the brain that are commonly affected in neurological diseases. GFAP, a marker of intermediate filaments in astrocytes that become hypertrophic in response to insult, has been shown to be increased in a number of neurological diseases, including NF1 [24]. Here, using a Dako anti-GFAP antibody (#70334) at 1:1000, we observed classic star-shaped GFAP^+^ immunolabeling in a 15-month-old *NF1* mutant miniswine (Fig 1A–1C, arrows) and in the thalamus of a 15-month-old wild-type miniswine (Fig 1D). The cytoplasmic localization pattern was similar to that seen previously in human and swine cerebrum [23, 25]. Importantly, a lack of immunopositivity was noted in neurons of the cortex (Fig 1A, asterisk) and granular cells of the cerebellum (Fig 1B, asterisk).

Injury and inflammatory factors activate ionized calcium binding adaptor molecule 1+ (Iba1) microglia in affected areas of the brain, such as the cerebellum of sheep exposed to LPS [26], and thalamus of wild-type mice exposed to traumatic brain injury [27]. Using a BioCare Medical anti-Iba1 antibody (#290) at 1:500, cytoplasmic Iba1 immunostaining was observed in both non-reactive, ramified microglia with numerous branching processes and more reactive amoeboid-like microglia (arrows) in the cortex, cerebellum and thalamus (Fig 1E–1G), with WT animals shown as a healthy control (Fig 1H). Filamentous immunopositive structures likely represent cross-sectional microglial processes, and the cytoplasmic localization is similar to published results in human and swine cerebrum (also referred to as AIf1) [23]. Iba1 immunolabeling appeared to be specific to microglia, as we saw no labeling in neurons in the cortex (Fig 1E, asterisk), to granular cells in the cerebellum (Fig 1F, asterisk), and to neurons in the thalamus (Fig 1G and 1H, asterisk).

Dysregulation of mature myelinating oligodendrocytes, which are labeled by myelin basic protein (MBP), can lead to loss of myelination. In other cases, a decrease in the density of oligodendrocytes in the prefrontal cortex may lead to schizophrenia, bipolar disorder, and major depressive disorder [28]. Using a Millipore anti-MBP antibody (#MAB386) at 1:100, large areas within the cortex, cerebellum and hippocampus were immunopositive for MBP (Fig 1I–1L, arrows). A higher magnified image of the tracts documents the filamentous morphology of the cytoplasmic and membranous localization of MBP to the myelin sheath (Fig 1I’–1L’). We see a similar localization pattern to the results shown in cerebral white matter of humans and swine [23]. There was no immunostaining of MBP to neurons and capillaries in the cortex (Fig 1I’, asterisk), to granule cells and capillaries in the cerebellum (Fig 1J’, asterisk) and to neurons in the hippocampus (Fig 1K’, asterisk) and thalamus (Fig 1L’, asterisk), indicating the specificity of this antibody to white matter tracts and its utility in swine tissue.

### Detection of reactive microglia and oligodendrocyte cell lineage markers

**Fig 2:**
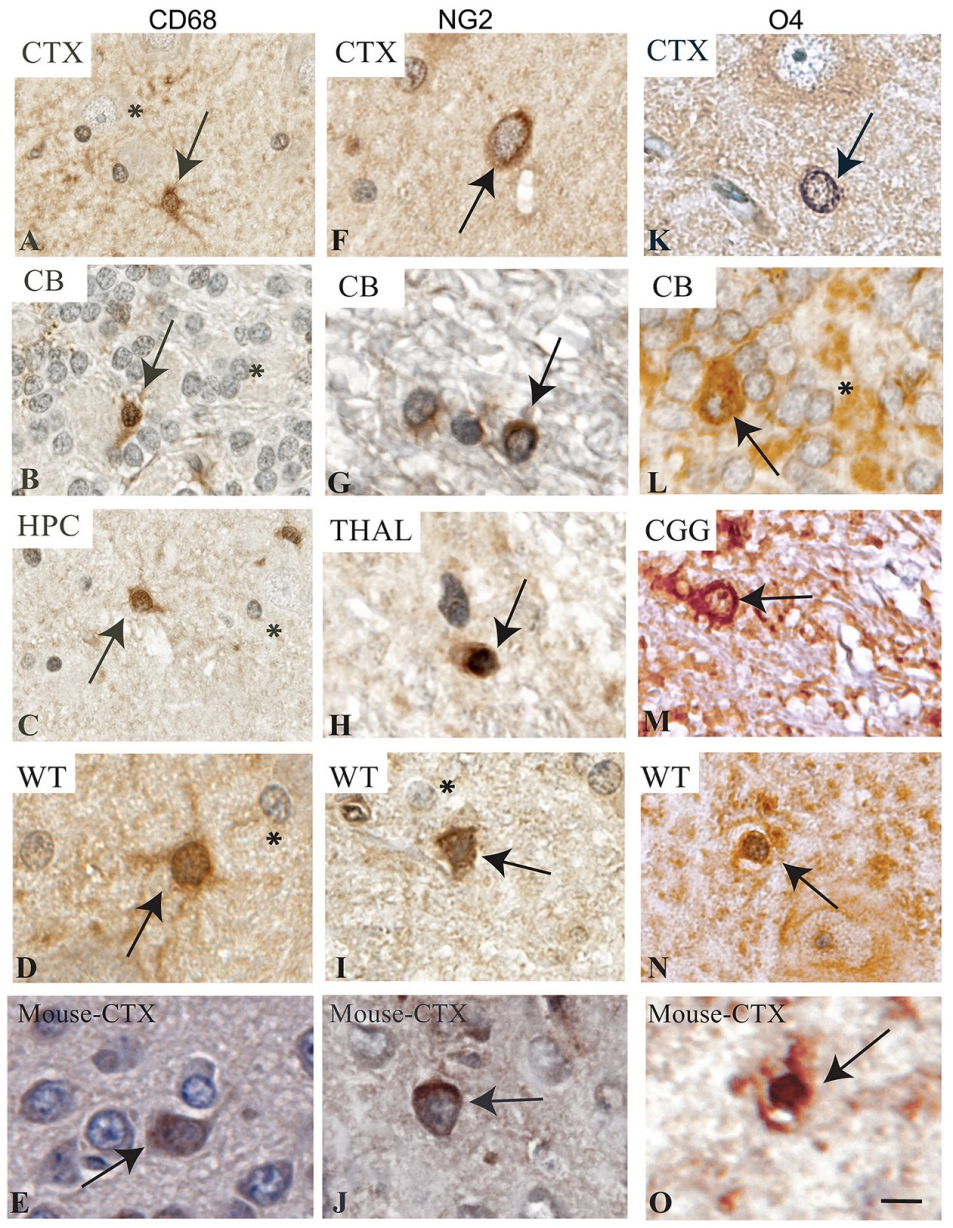
Immunolabeling of microglial and pre-oligodendrocyte markers in the *NF1* mutant and wild-type miniswine brain. Brain regions are identified as cortex (CTX), cerebellum (CB), thalamus (THAL), hippocampus (HPC) and cerebral aqueduct (CGG). A-D: CD68+ expression in the cortex, cerebellum, hippocampus, and thalamus, localized to the membrane of microglia. Arrows indicate ameboid-like microglia. E: CD68+ expression in mouse cerebral cortex. F-I: NG2+ expression in the cortex, cerebellum, and thalamus, localized to the membrane and cytoplasm of oligodendrocyte progenitors. Arrows indicate immature oligodendrocytes. J: NG2+ expression in mouse cerebral cortex. K-N: O4+ expression in the cortex, cerebellum, cerebral aqueduct, and thalamus localized to the membrane of pre-oligodendrocytes. Arrows indicate pre-oligodendrocytes. O: O4+ expression in mouse cerebral cortex. Asterisks indicate lack of immunolabeling. Scale bar: 50μm. WT animals shown as a healthy control.

CD68 immunoreactivity has been documented in microglia within the cerebral cortex of humans [25], and in the multiple brain regions of aging wild-type mice and mice exposed to an neurological insult such as LPS [29]. Here reactive microglia were indentified using an Abcam anti-CD68 antibody (#ab125212) at 1:400, showing localization to the cytoplasm (arrows) in the cortex, cerebellum, hippocampus, and thalamus (Fig 2A–2D). We observed a similar cytoplasm localization in mouse cerebral cortex (Fig 2E). We tested multiple dilutions with this antibody and found the least background staining with a 1:400 dilution. At this dilution, we did not see immunostaining of CD68 to neurons in the cortex (Fig 2A, asterisk), to granular cells in the cerebellum (Fig 2B, asterisk), or to neurons in the hippocampus (Fig 2C, asterisk) or thalamus (Fig 2D, asterisk).

Tracing cell type lineage can be incredibly informative in determining mechanisms of disease and appropriate intervention points. Oligodendrocytes have a well-studied lineage and are particularly important for the pathology of NF1. For example, oligodendrocyte precursor cells (OPCs) have been identified as the cell of origin for gliomas in *Nf1* conditional knockout mice [30], with *Nf1* mutant mice expressing more OPCs in the brain. As expected, we observed membrane and cytoplasmic neural/glial antigen 2+ (NG2) immunostaining in the cortex, cerebellum and thalamus when using an Abcam anti-NG2 antibody (ab129051) at 1:250, indicating the presence of oligodendrocyte progenitors (Fig 2F–2I, arrows). A similar membrane and cytoplasmic localization was seen in the mouse cerebral cortex (Fig 2J), consistent with previous reports of NG2+ immunostaining throughout the rodent brain and in the cerebral cortex and cerebellum in human tissues [25, 31]. Similarly, oligodendrocyte marker 4+ (O4+) immunostaining, a marker for pre-oligodendrocytes, has been found in the corpus callosum in young rat pups [32], in the rat cerebellum [33], and in the midbrain (substantia nigra pars compacta) of control and neurotoxin exposed C57BL/6 mice [34]. O4 immunostaining via an R&D Systems anti-O4 antibody (#MAB1326-SP) at 1:400 showed localization to the membrane, indicating the presence of pre-oligodendrocytes in the cortex, cerebellum, cerebral aqueduct, and thalamus (Fig 2K–2N, arrows). Compared to mouse cerebral cortex, we see a similar membrane localization pattern (Fig 2O), and importantly we did not see immunostaining of O4 in the granular cells of the cerebellum (Fig 2L, asterisk).

### Detection of mature oligodendrocytes in miniswine tissue

**Fig 3:**
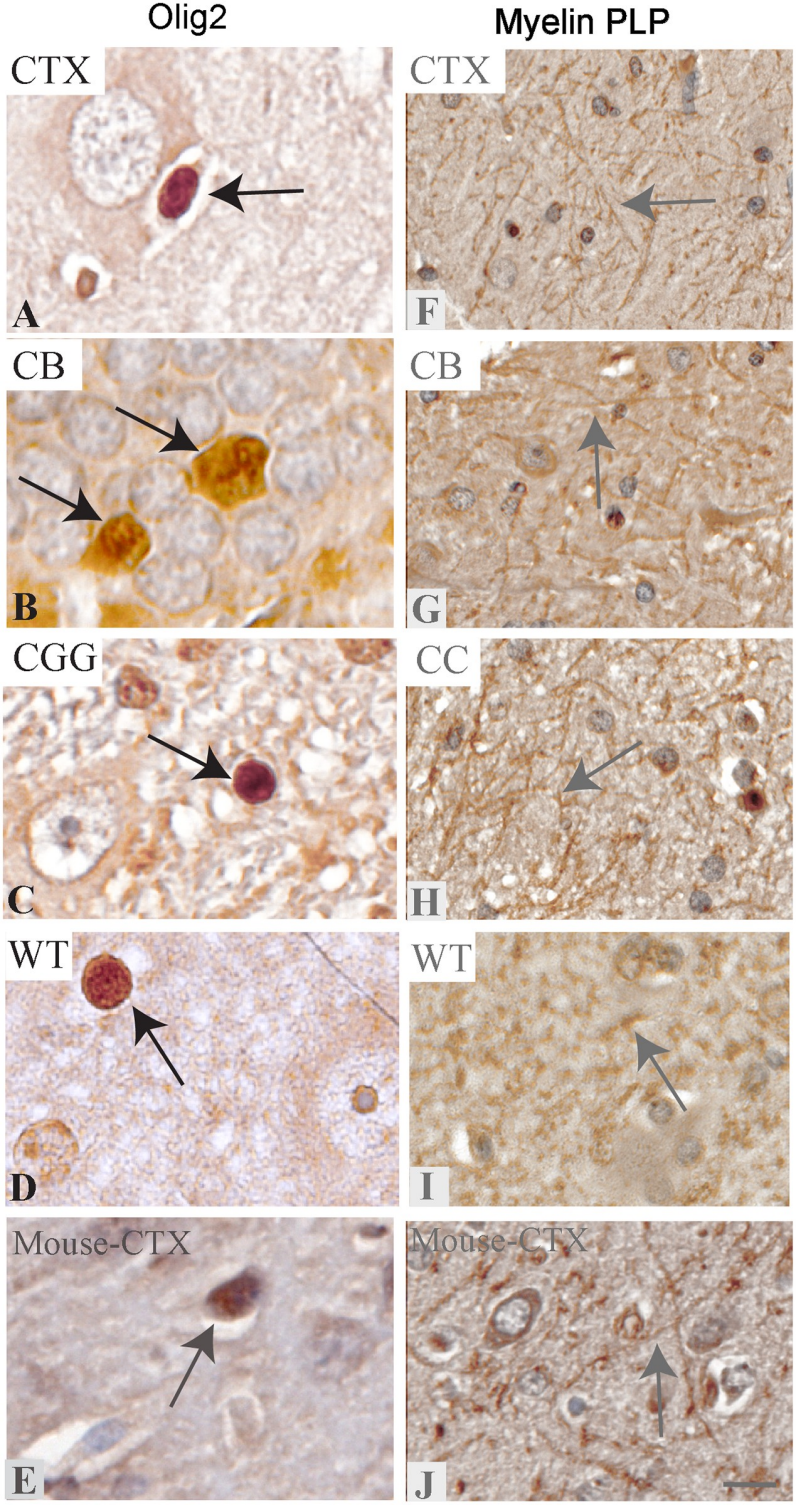
Immunolabeling of mature oligodendrocyte lineage markers in the *NF1* mutant and wild-type miniswine brain. Brain regions are identified as cortex (CTX), cerebellum (CB), cerebral aqueduct (CGG) and corpus callosum (CC). A-D: Olig2+ expression in the cortex, cerebellum, cerebral aqueduct, and thalamus in wild-type, localized to the nucleus and cytoplasm of oligodendrocytes. Arrows indicate mature oligodendrocytes. E: Olig2+ expression in mouse cerebral cortex. F-I: Myelin PLP+ expression in the cortex, cerebellum, corpus callosum, and thalamus in wild-type localized to the membrane and cytoplasm of multiple cells as well as to the filamentous structures of the neuropil. Non-specific staining to neurons as well as background staining was found. J: Myelin PLP+ expression in mouse cerebral cortex. Asterisks indicate lack of immunolabeling. WT animals shown as a healthy control. Scale bar: 50μm.

Olig2 is a pan-oligodendrocyte marker most robustly expressed in immature oligodendrocytes of the developing brain [35]. In the adult human brain, Olig2^+^ cells can be found in the cerebral cortex, in molecular and granular layer cells of the cerebellum [25], and in similar regions in mice [36, 37]. Using a Millipore anti-Olig2 antibody (#ab9610) at 1:500, the nucleus and cytoplasm of multiple oligodendrocytes were immunopositive for anti-Olig2 antibodies in the cortex, cerebellum, cerebral aqueduct and thalamus (Fig 3A–3D, arrows). We noted lack of immunopositivity of Olig2 in neurons of the cortex as an internal negative control (Fig 3A, asterisk). Comparatively, we see evidence of nuclear immunostaining in mouse cerebral cortex (Fig 3E). As a marker of mature oligodendrocytes, we also tested a myelin proteolipid protein (Myelin PLP) antibody (Abcam #ab28486 at 1:500) with inconsistent results. There was faint membrane expression in the white matter tracts of the cortex (Fig 3F), cerebellum (Fig 3G) corpus callosum (Fig 3H) and thalamus (Fig 3I) to the neuropil, though there appears to be non-specific staining in other cells within the CNS. We see similar staining to the neuropil and non-specific immunostaining in mouse cerebral cortex (Fig 3J). The neuropil and membrane immunostaining are similar to the localization pattern as shown in the Abcam datasheet, however, the non-specific staining in neurons and background staining in our images indicate that we have not optimized the protocol for this antibody.

### Immunolabeling validation of neuronal subtypes and neurotransmitters

Spatial learning and memory deficits are a prominent feature of neurological disease, specifically affecting dopamine, GABA, and glutamate signaling in hippocampal neurons [15, 38]. Dysregulated GABA signaling in the CNS, which causes an increase of GABA-mediated inhibition, has been implicated as a cause of learning defects in mouse models of Huntington’s disease and NF1 [15, 39]. Loss of dopamine signaling in hippocampal neurons has been proposed to be a reason for the spatial learning and memory defects in mutant *Nf1* mutant mice [38]. As such, proper neuronal placement, maturation, and signaling is crucial for proper CNS function, indicating the need for robust neuronal antibodies in miniswine tissue.

**Fig 4:**
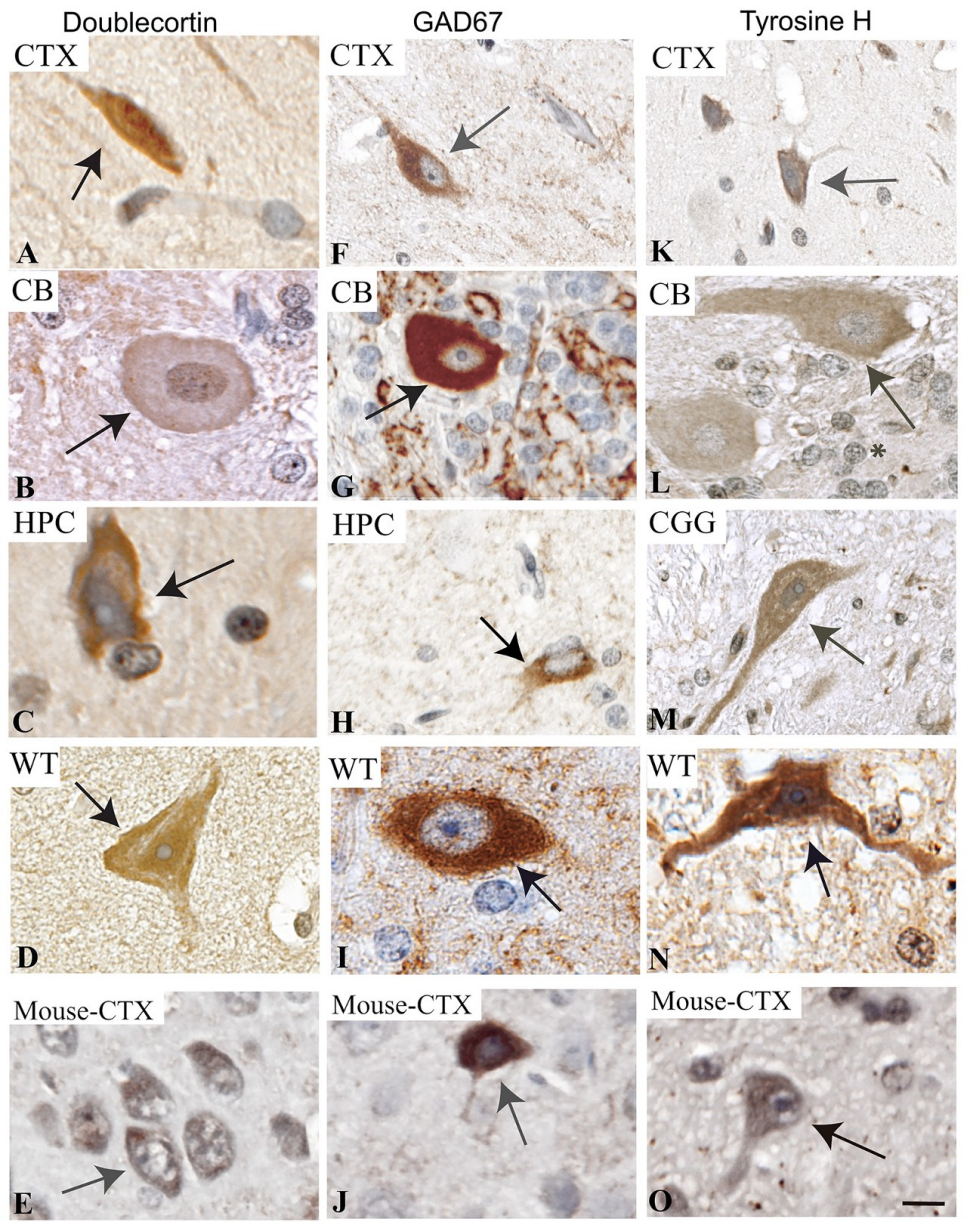
Immunolabeling of neuron markers in the *NF1* mutant and wild-type miniswine brain. Brain regions are identified as cortex (CTX), cerebellum (CB), hippocampus (HPC) and cerebral aqueduct (CGG). A-D: Doublecortin+ expression in the cortex, cerebellum, hippocampus, and cerebral aqueduct in wild-type miniswine, localized to the cytoplasm of neurons. Arrows indicate immature neurons, with minimal staining in the cerebellum. E: Doublecortin+ expression in mouse cerebral cortex. F-I: GAD67+ expression in the cortex, cerebellum, hippocampus, and thalamus in wild-type localized to the cytoplasm of neurons. Arrows indicate GABAergic neuron cell bodies. J: GAD67+ expression in mouse cerebral cortex. K-N: Tyrosine H+ expression in the cortex, cerebellum, and cerebral aqueduct in *NF1* and wild-type miniswine, localized to the cytoplasm of neurons. Arrows indicate dopaminergic neurons. O: Tyrosine H+ expression in mouse cerebral cortex. Asterisks indicate lack of immunolabeling. Scale bar: 50μm.

Doublecortin (DCX), a marker of immature neurons, has been documented in the cerebral cortex and hippocampus in mice [40], and in the granular cell layer of the adult rat cerebellum [41]. Using a SantaCruz anti-DCX antibody (#sc-28939) at 1:50, we observed doublecortin (DCX)^+^ immunolabeling in the cortex, cerebellum, hippocampus and cerebral aqueduct of the adult miniswine brain, indicating the presence of immature neurons (Fig 4A–4D). Cytoplasmic immunostaining of DCX was also found in mouse cerebral cortex (Fig 4E).

Inhibitory interneurons that express GAD67 are small and /or medium-sized oval shaped cells with cytoplasmic expression in the cell body and dendritic processes (48). Very clear immunostaining with a BD BioSciences anti-GAD67 antibody (#611604) at 1:50 was found in the cytoplasm of these neurons, specifically localized to the cell bodies (Fig 4E–4G; single arrow) in the cortex, cerebellum, and hippocampus of the *NF1* miniswine brain (Fig 4F–4I) and thalamus of wild-type brain (Fig 4I). GAD67+ neurons have also been documented within these brain regions in humans [25]. We see similar cytoplasmic and membranous immunostaining of GAD67 in mouse cerebral cortex (Fig 4J). Similarly, prominent cytoplasmic tyrosine hydroxylase immunostaining, a marker of dopaminergic neurons, was found in the cytoplasm of neurons in the cerebral aqueduct, cortex and Purkinje cells of the cerebellum (Fig 4K–4N) when using a Millipore anti-TH antibody (#ab152 at 1:500). We did not see immunostaining to the granular cells in the cerebellum (Fig 4L, asterisk). Immunolabeling matched the cytoplasmic localization of tyrosine hydroxylase in mouse cerebral cortex (Fig 4O). The immunoreactivity of tyrosine hydroxylase is as expected, as this marker has been found to immunoreact to the cerebellar lobules and Purkinje cells of the cerebellum in adult mice [42].

### Immunolabeling of free-floating sections

In larger animals, formalin fixation and cryopreservation of larger blocks of tissue enables the sectioning of whole brain regions [43]. Blocks of tissue can be easily sectioned on a freezing microtome, thick sections placed into well plates, and IHC can proceed with only an endogenous peroxidase block before primary antibody incubation. Formalin-fixed paraffin embedded (FFPE) tissue requires greater processing as antigen retrieval is necessary to allow the epitopes to be available for the antigens, hence more steps are required, and more troubleshooting may be necessary than formalin-fixed frozen tissue [44]. Free-floating sections may also provide clearer results for future quantitative analyses as less background staining occurs compared to FFPE sections. Hence, we tested antibodies where we found with higher levels of background staining using FFPE in our hands (GFAP, CD68, Olig2, doublecortin, GAD67, and tyrosine hydroxylase) using free-floating section techniques instead (S1 Fig). Using this method, the level of background staining was reduced for GFAP in the hippocampus of a 20 month old, male *NF1* miniswine. GFAP+ immunostaining to the membrane and to the intermediate filaments of astrocytes was observed (S1 Fig). The remaining antigens were immunolabeled to free-floating sections in a 14 month old, female *NF1* miniswine. For the CD68 antibody, we did not see any background staining in the thalamus. The CD68 antibody localized to the membrane and to a few ramified processes (S1 Fig). Similarly, the background was almost absent in the cerebellum sections that were immunolabeled with Olig2, and we observed very clear nuclear immunolabeling (S1 Fig). Only a few neurons were immunopositive for doublecortin in the cerebellum, but we did observe cytoplasmic immunostaining and no background staining (S1 Fig). Multiple neurons were immunopositive for GAD67 in the thalamus, localizing to the cytoplasm and neuropil of the thalamic neurons (S1 Fig). Neuropil has a dot-like and filamentous appearance, which is expected, and can be distinguished from the diffuse appearance of background staining. We see a similar filamentous pattern of neuropil immunopositivity with the tyrosine hydroxylase antibody in the thalamus of the *NF1* miniswine (S1 Fig). Therefore, free-floating techniques may be offer cleaner results when compared to standard FFPE techniques, and the use of free-floating sections may be suggested for future studies involving swine immunohistochemistry.

### Nociceptive markers tentatively identified in dorsal root ganglion

**Fig 5:**
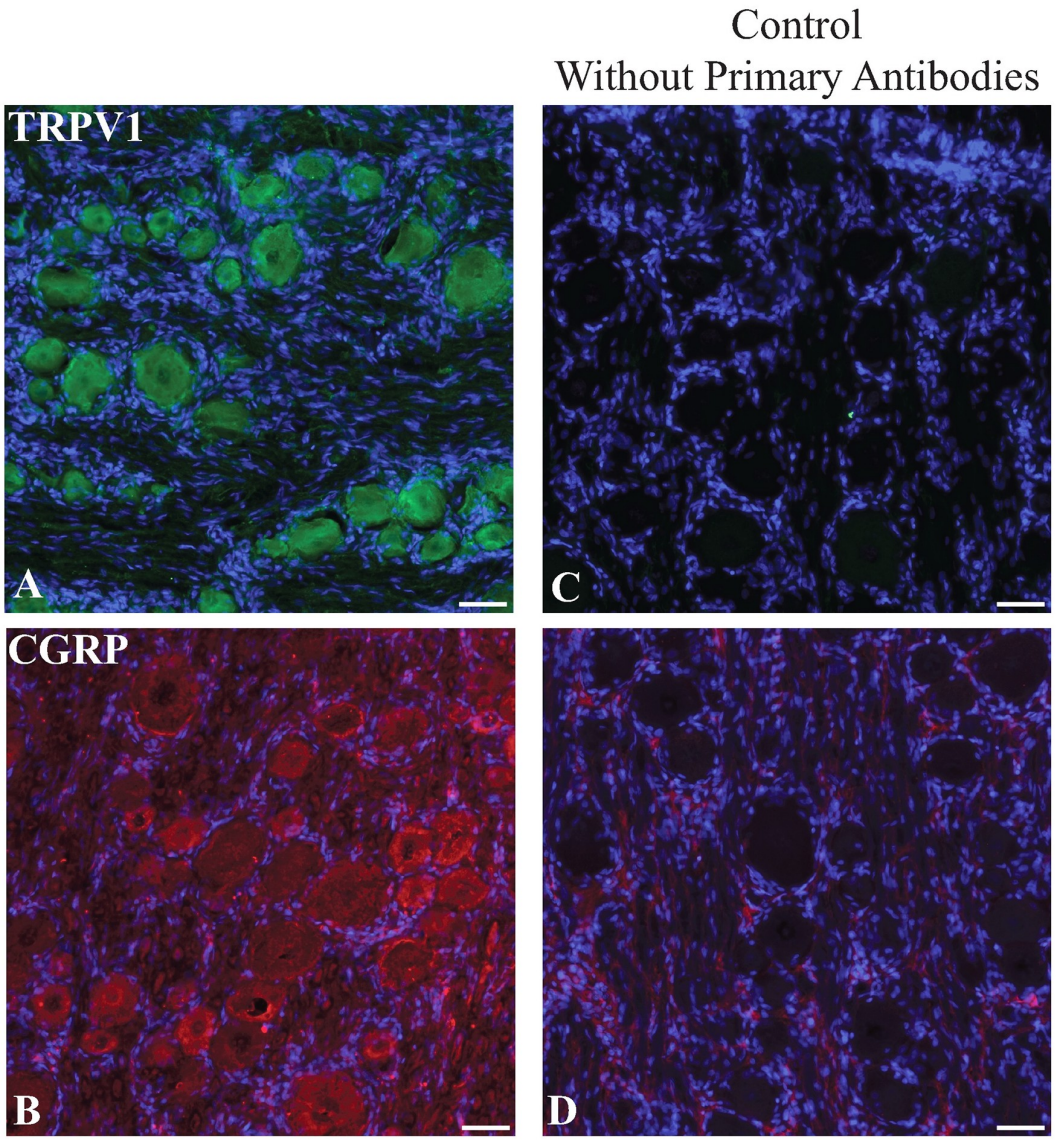
Immunolabeling miniswine DRGs. A-B: Miniswine DRGs were immunostained with commercial antibodies against TRPV1 and CGRP. C-D: Negative controls omitting primary antibodies. Nuclei were counterstained with DAPI. Scale bars: 50μm.

Chronic pain is usually a component of many neurological diseases that affects approximately 20–40% of patients in general [45]. We investigated the utility of pain perception-nociceptive markers in dorsal root ganglion isolated from our mutant miniswine. Sliced DRGs from miniswine were immunostained with antibodies raised against calcitonin gene related peptide (CGRP), a pro-nociceptive neurotransmitter, and transient receptor potential cation channel subfamily V member 1 (TRPV1), a capsaicin receptor. Unlike immunostaining patterns observed in rodents and Rhesus monkeys, in which subpopulations of neurons were stained by each antibody [46, 47]; all DRG neurons were stained by antibodies against CGRP and TRPV1 with different fluorescent intensities in miniswine (Fig 5A and 5B). For one of the antibodies, differences in staining pattern were observed between batches. Control omitting primary antibodies revealed no non-specific staining due to the secondary antibodies (Fig 5C and 5D). However, comparison of the immunogen sequences with swine (Sus scrofa) protein databases in S1 Table, revealed that several off-target proteins could also bound by these antibodies. Therefore, better controls are necessary to validate these antibodies for use in swine.

## Discussion

Models of disease, especially in swine, need validated and reproducible experimental tools for effective study and development of translational therapies. Research into neurological diseases that have already been modeled in transgenic swine, including Huntington’s [48, 49]; Alzheimer’s [50–52]; Parkinson’s [53, 54]; ALS [55, 56] and Neurofibromatosis type-1 [3, 57], would benefit from verifiable antibodies that are specific to swine CNS cell antigens. A huge challenge in developing more translatable large animal models of diseases will persist if appropriate reagents are not readily available. For example, while a particular antibody of interest may be available, they are more likely to react in humans or mice, requiring significant time and resources to optimize immunostaining conditions in porcine tissue. Resources to test numerous antibodies is often limited and, ultimately, because of the lack of validated tools, it can place technical constraints on proper experimental design, making accessible resources such as this study critical in the swine model community. Here, we validated a number of CNS relevant antibodies in the miniswine brain, focusing on neuronal and glial targets. In particular, we found many antibodies that consistently labeled the expected cell type and targeted the appropriate cellular compartment, while simultaneously being absent from unexpected cell types. Additionally, these antibodies were able to label their anticipated targets throughout multiple tissue sections, across multiple occasions, using FFPE and formalin fixed cryopreserved tissue with slightly different immunohistochemical techniques (slide stained vs free-floating), indicating the specificity of these reagents.

The glial targets that we focused upon play an important role in the pathogenesis of neurological diseases, especially for documenting neuroinflammation (Iba1+ and CD68+ microglia), astrocytosis (GFAP+ astrocytes), and disruption of myelination (NG2+ and O4+ OPCs, MBP and myelin PLP+ mature oligodendrocytes, Olig2+ all oligodendrocytes). These glial markers are widely studied in Huntington’s and Parkinson’s disease [58], multiple sclerosis [59], amyotrophic lateral sclerosis-ALS [58, 60] and Alzheimer’s disease [58, 60–62] as their expression changes in response to activation or differentiation. Activated glia may either become neuroprotective or neurotoxic, depending on the cytokines, chemokines, or growth factors that are released [63]. If neurotoxic, disruption in neuronal signaling and neuronal loss can lead to a number of neurological deficits including cognitive impairment and motor dysfunction. The neuronal markers that we validated here for GABAergic (GAD67+) and dopaminergic (tyrosine hydroxylase+) neurons, have been used to document imbalanced signaling associated with learning and memory deficits in NF1 [15, 38, 64] and with other cognitive and motor impairments in Huntington’s disease [39]. Utilizing these markers to study mechanisms of glial activation and differentiation, and mechanisms of neuronal loss and transmission during the pathogenesis of neurological diseases will help to develop therapeutics that treat and prevent these diseases.

Ours is the first study validating antibodies specific for NG2, Olig-2, GAD67, and tyrosine hydroxylase in the CNS of mutant and wild-type miniswine; and the first study validating GFAP, Iba1, MBP, and doublecortin in the cerebral cortex, cerebellum, thalamus, and hippocampus of miniswine. Several studies of swine models of CNS injuries/disorders have tested the specificity of antibodies on nervous tissue [65–69]. However, two of these have focused solely on the spinal cord and not the brain itself [66, 67]. Of the remaining studies, only one of these describes the impact of CNS injuries/disorders by addressing axonal injury and astrocytic/microglial re-activity [65]. The other brain immunohistological study was similar to ours as it validated antibodies, however this validation was compared to a general histological stain (Giemsa) [68]. Moreover, we recently published a comprehensive study on antibody immunoreactivity in non-CNS swine tissues, (GFAP, Iba1, and MBP in the cerebrum) specifically wild-type swine [23], but we determined that further study was necessary to validate markers in genetically modified swine that recapitulate characteristics of a human disease. We accomplished this validation through IHC, which limits our study, however this technique is one of the most frequently used metrics of protein expression [70] due to its relative ease of use and low cost. The markers in this study have been highly explored in mice models and negative and positive controls have been identified, which enhances the reliability of IHC for verification purposes. However, the use of western blotting on brain tissue lysates would add another level of validation, and we intend to utilize this method in future publications.

Though we were able to validate all these markers in various regions of mutant and wild-type miniswine brains, we only tested nociceptive markers in peripheral CNS tissues (i.e. spinal cord). Neurological diseases such as amyotrophic lateral sclerosis (ALS) have neuronal loss and degeneration in both the brain and spinal cord. Validating CNS markers in the swine spinal cord would improve the utility of swine models that investigate neurodegeneration throughout the CNS. Moreover, we only validated markers of dopaminergic and GABAergic neurotransmission in this study, thus further work to validate markers specific for glutamatergic neurons (excitatory), serotonergic neurons (cognition, learning, memory), and cholinergic neurons (acetylcholine-motor) in miniswine models is still needed. In conclusion, validation of immunological tools (most commonly tested only on rodent or human tissue) in swine tissues will improve the clinical and translational aspects of the swine model for disease research. The validation of the antibodies described in this paper provides new tools that will aid in the further investigation of the role of neurofibromin in neurological function, as well as other swine models of neurological diseases.

## Supporting information

S1 Fig Immunolabeling of antibodies to free-floating sections in *NF1* miniswine. Brain regions are identified as hippocampus (HPC), thalamus (THAL) and cerebellum (CB). A: Immunolabeling of GFAP to the membrane and filaments of astrocytes in the hippocampus of a 20-month old male *NF1* miniswine. The following images are from a 14-month old female *NF1* miniswine. B: Immunolabeling of CD68 to the membrane and ramified processes of microglia in the thalamus. Note that lack of background staining. C: Immunolabeling of Olig2 to the nucleus of oligodendrocytes in the cerebellum. D: Immunolabeling of doublecortin to the cytoplasm of neurons in the thalamus. E: GAD67+ immunostaining to the cytoplasm and neuropil of thalamic neurons. Note the dot-like and filamentous appearance of the neuropil surrounding each neuron. F: Immunostaining of tyrosine hydroxylase to neuropil in the thalamus. Scale bar 10μm.
(TIF)

S2 Fig ARRIVE Guidelines Checklist. Checklist detailing the location of specific items in the Title, Abstract, Introduction, Methods, Results, and Discussion of the publication: Validating indicators of CNS disorders in a swine model of neurological disease.
(PDF)

S1 Table Sequence identify between the immunogen of the antibody to a porcine protein and methods of antibody validation. The Antibody, immunogen details, immunogen accession, BLAST sequence identifier, % identity to the porcine protein, cross reactive proteins indicated by BLAST at % identity ≥ 55% and query coverage ≥ 50%, how the antibody was validated by the company, and citations relevant to each antibody are listed in the table. The immunogen details (when available) include the amino acid sequence used to develop the immunogen and the animal that was immunized with the immunogen. The immunogen accession is the NCBI accession number and the BLAST sequence ID is the NCBI accession number for the porcine protein. Antibodies were validated according to their respective company for the following techniques: WB (western blot), IHC (immunohistochemistry), ICC (immunocytochemistry), IP (immunoprecipitation), ICC/IF (immunocytochemistry-immunofluorescence), Flow Cyt (flow cytometry), CyTOF (mass cytometry) and ELISA. The citation either refers to the company’s webpage for each antibody or the accession number in The Antibody Registry (antibodyregistry.org). * Indicates the antibody was found in The Antibody Registry. # Indicates cross reactive proteins identified by % identity ≥80% and query coverage ≥ 50%.
(PDF)
