# Supplementary figures and images for: Validating indicators of CNS disorders in a swine model of neurological disease
Source: PLoS One. 2020 Feb 19;15(2):e0228222. doi: 10.1371/journal.pone.0228222 (PMC7029865; doi:10.1371/journal.pone.0228222)

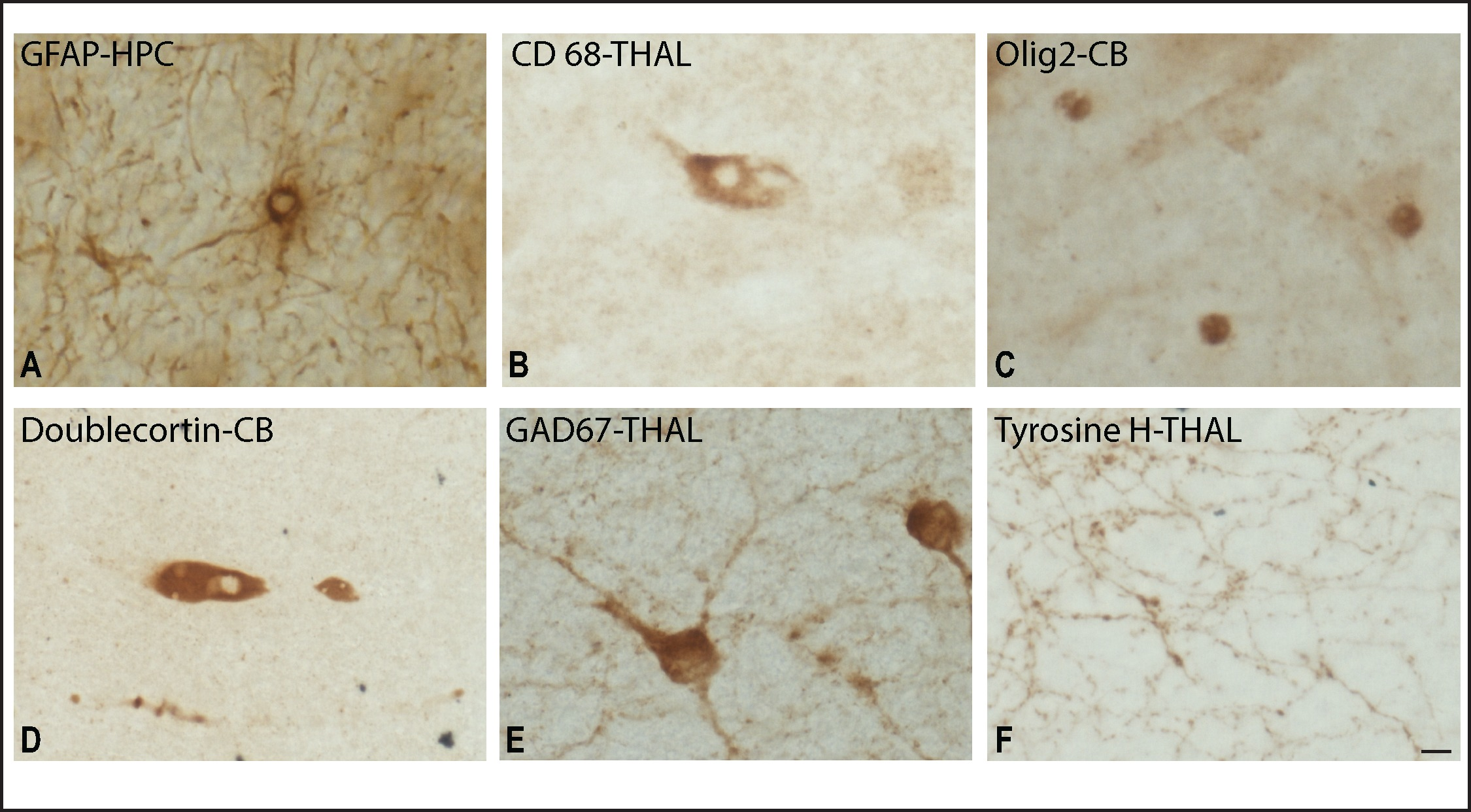

Supplement: S1 Fig — Brain regions are identified as hippocampus (HPC), thalamus (THAL) and cerebellum (CB). A: Immunolabeling of GFAP to the membrane and filaments of astrocytes in the hippocampus of a 20-month old male NF1 miniswine. The following images are from a 14-month old female NF1 miniswine. B: Immunolabeling of CD68 to the membrane and ramified processes of microglia in the thalamus. Note that lack of background staining. C: Immunolabeling of Olig2 to the nucleus of oligodendrocytes in the cerebellum. D: Immunolabeling of doublecortin to the cytoplasm of neurons in the thalamus. E: GAD67+ immunostaining to the cytoplasm and neuropil of thalamic neurons. Note the dot-like and filamentous appearance of the neuropil surrounding each neuron. F: Immunostaining of tyrosine hydroxylase to neuropil in the thalamus. Scale bar 10μm. (TIF) [file pone.0228222.s001.tif]
