# Supplementary material for: Validating indicators of CNS disorders in a swine model of neurological disease
Source: PLoS One. 2020 Feb 19;15(2):e0228222. doi: 10.1371/journal.pone.0228222 (PMC7029865; doi:10.1371/journal.pone.0228222)
Supplement: S1 Table — The Antibody, immunogen details, immunogen accession, BLAST sequence identifier, % identity to the porcine protein, cross reactive proteins indicated by BLAST at % identity ≥ 55% and query coverage ≥ 50%, how the antibody was validated by the company, and citations relevant to each antibody are listed in the table. The immunogen details (when available) include the amino acid sequence used to develop the immunogen and the animal that was immunized with the immunogen. The immunogen accession is the NCBI accession number and the BLAST sequence ID is the NCBI accession number for the porcine protein. Antibodies were validated according to their respective company for the following techniques: WB (western blot), IHC (immunohistochemistry), ICC (immunocytochemistry), IP (immunoprecipitation), ICC/IF (immunocytochemistry-immunofluorescence), Flow Cyt (flow cytometry), CyTOF (mass cytometry) and ELISA. The citation either refers to the company’s webpage for each antibody or the accession number in The Antibody Registry (antibodyregistry.org). * Indicates the antibody was found in The Antibody Registry. # Indicates cross reactive proteins identified by % identity ≥80% and query coverage ≥ 50%. (PDF) [file pone.0228222.s003.pdf]

Antibody	Immunogen details	Immunogen accession	BLAST sequence ID	% Identifies	CR Proteins w % ID ≥55; QC ≥50	Validated by	Citations
GFAP-Dako z0334	Bovine origin	Q28115.2	NP_001231326.1	96%	Vimentin, Desmin, Peripherin	WB, IHC, ICC, IP	www.labome.com/product/Dako/Z0334.html
CD 68-ab125212	AA 312-326 of Mouse origin	P31996	NP_001278705.1	69%	LPTS-RP2, LRP-2[#]	WB, IHC	www.abcam.com/cd68-antibody-ab125212.html
IBA1-BioCare Medical 290	The full length of peptide	NA	NA	NA	NA	IHC	biocare.net/product/microglia-antibody/
NG2-ab129051	AA 300-325 of Mouse origin	Q8VHY0	XP_003128533.4	92%	0	WB, IHC, ICC/IF	www.abcam.com/ng2-antibody-ab129051.html
O4-R&D Systems MAB1326	Bovine origin	NA	NA	NA	NA	Flow Cyt, CyTOF, ICC	www.rndsystems.com/products/human-mouse-rat-chicken-oligodendrocyte-marker-o4-antibody-o4_mab1326
Olig2-Millipore ab9610*	Whole recombinant of Mouse origin	NP_058663	XP_003358977.2	96%	Olig3	WB, IP, IHC	AB_570666 in The Antibody Registry; antibodyregistry.org
MBP-Millipore MAB386*	AA 82-87 of Bovine origin	NP_001193603.1	NP_001001546.1	100%	DDHD2, Nucleoporin Nup205, 4-PH alpha-1[#]	WB, ELISA, IHC, RIA	AB_94975 in the Antibody Registry; http://antibodyregistry.org
Doublecortin-sc28939	AA 162-441 of human origin	NP_000546.2	XP_013841791.1	99%	Doublecortin-like kinase1	WB, ELISA, IP, IF	www.scbt.com/scbt/product/doublecortin-antibody-h-280
GAD67-BDBioSciences 611604	AA 17-130 of rat origin	NP_058703	NP_999059.1	95%	0	WB	BD Biosciences spec sheet on WB
Tyrosine H-Millipore ab152	Full length peptide of Rat origin	AAA42258	XP_020941111.1	85%	0	WB, ELISA, IF, IHC, IP	www.emdmillipore.com/US/en/product/Anti-Tyrosine-Hydroxylase-Antibody,MM_NF-AB152
Myelin PLP-ab28486	AA 109-128 of Mouse origin	P60202	NP_999139.1	100%	RRP[#]	WB, ELISA, IHC, ICC, Flow Cyt, ICC/IF	www.abcam.com/myelin-plp-antibody-ab28486.html
TRPV1-Neuromics GP14100*	AA 760-781 of Rat origin	NP_114188.1	XP_013836670.2	68%	Transcription elongation factor A protein 2[#]	IHC	AB_2209002 in the Antibody Registry; antibodyregistry.org
CGRP-abcam 36001*	AA 23-37 of Rat origin	NP_001029128.1	NP_001095943.1	87%	Calcitonin gene-related peptide 2 isoform 2 precursor[#]	IHC, ELISA	AB_725807 in the Antibody Registry; antibodyregistry.org
# ≥80% ID							
